# MKP‐1 regulates the inflammatory activation of microglia against Alzheimer's disease

**DOI:** 10.1111/cns.14409

**Published:** 2023-08-21

**Authors:** Junhua Li, Lin Wang, Qinhua Zeng, Jing He, Qing Tang, Kejian Wang, Guiqiong He

**Affiliations:** ^1^ Institute of Neuroscience, Basic Medical College Chongqing Medical University Chongqing China; ^2^ Department of Anatomy, Basic Medical College Chongqing Medical University Chongqing China; ^3^ Department of Basic Medicine Chongqing College of Traditional Chinese Medicine Chongqing China

**Keywords:** Alzheimer's disease, microglia, MKP‐1, neuroinflammation

## Abstract

**Background:**

Alzheimer's disease (AD) is one of the most common neurodegenerative diseases leading to dementia in elderly people. Microglia‐mediated neuroinflammation plays an important role in AD pathogenesis, so modulation of neuroinflammation has emerged as an essential therapeutic method to improve AD. The current study aims to investigate whether MKP‐1 can regulate microglia phenotype and inflammatory factor release in AD and explore its possible mechanisms.

**Methods:**

Amyloid precursor protein/PS1 double transgenic mice and wild‐type mice were selected to study the locations of microglia and amyloid‐β (Aβ) plaques in different regions of mice brains. Changes in MKP‐1 of microglia were detected using AD model mice and AD model cells. Changes in phenotype and the release of inflammatory factors within immortalized BV2 murine microglia were investigated by regulating the expression of MKP‐1.

**Results:**

The distribution of microglia and Aβ plaques in the AD brain was region‐specific. MKP‐1 expression was downregulated in AD mice, and in vitro, with increasing Aβ concentrations, MKP‐1 expression was reduced. MKP‐1 over‐expression increased M2 microglia but decreased M1 microglia accompanied by changes in inflammatory factors and inhibition of MKP‐1 yielded the opposite result.

**Conclusion:**

MKP‐1 regulated microglia phenotype and inflammatory factor release in AD through modulation of the p38 signaling pathway.

## Graphical abstract

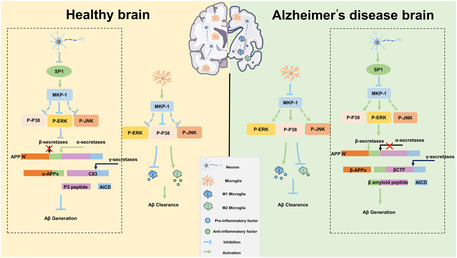

## 1 INTRODUCTION

Alzheimer's disease (AD) is an insidious and progressive degenerative disease of the nervous system. Numerous studies suggest that AD is not attributable to any single factor but involves multiple genetic, environmental, health conditions, and lifestyle facets.^1^ AD pathogenesis is known to be typified by the accumulation of extracellular amyloid‐β (Aβ) plaques, intracellular neuronal fiber tangles (NFTs), neuronal and synaptic regression accompanied by neuroinflammation^2^, ^3^; however, development of the disease remains ill‐defined. One plausible explanation for the pathogenesis of AD relates to the amyloid cascade hypothesis. Under normal conditions, the amyloid precursor protein (APP) is converted into soluble APPα fragments catalyzed by α‐secretase whereas APP is converted into Aβ catalyzed by β‐secretase and γ‐secretase during the pathological state.^4^ Aβ is then considered to induce microglia activation and neuroinflammation, which alongside the release of inflammatory mediators from microglia, facilitates the production and accumulation of Aβ.^5^ This process culminates in a noxious circle of inflammation created amongst Aβ aggregates, activated microglia, and microglia inflammatory mediators to further enhance Aβ deposition and neuroinflammation. In essence, neuroinflammation plays an irrevocable role in the development of AD.

Neuroinflammation is characterized by a proliferation of reactive glial cells around amyloid plaques,^6^ in which physiologically normal microglia appear as “resting” entities in the brain are transformed or “activated” when damage (injury or stress) is detected.^7^ Activated microglia are divided into the classical, M1‐state and the selectively activated M2‐state phenotypes.^8^ In the M1‐state, pro‐inflammatory cytokines are released to enhance inflammatory reactions but anti‐inflammatory cytokines with the ability to phagocytose cellular debris and suppress immune inflammatory responses are secreted from M2 microglia.^9^ Neuroinflammation is also characterized by changes in the levels of several cellular components including inducible nitric oxide synthase^10^ (iNOS), tumor necrosis factor‐alpha^11^ (TNF‐α), Arginase 1^12^ (Arg1), and triggering receptor expressed on myeloid cells 2^13^ (Trem2) proteins. The ratio of M1 to M2 microglia is a pre‐requisite of neuroinflammation, since M2 microglia predominate during the initial stages of inflammation, being involved in the clearance of amyloid plaques.^14^ As inflammation proceeds, the proportion of M1 type surpasses M2 leading to dysregulation of microglia phenotype as AD progresses.^15^ Moreover, the genetic switch from anti‐inflammatory M2 phenotype to pro‐inflammatory M1^16^ has been suggested to be an underlying mechanism for the release of additional pro‐inflammatory factors that contribute accelerated progression of AD.^6^ Thus, the full understanding of the molecular mechanisms required for microglia activation and neuroinflammation would undoubtedly be of significant clinical benefit to sufferers of AD.

Recent studies have shown that mitogen‐activated protein kinase (MAPK) phosphatase‐1 (MKP‐1), an archetypal member of the MAPK dual‐specificity phosphatase family, is required for the regulation of macrophage phenotype and functions.^17^ Data from the human protein atlas indicate that MKP‐1 is constitutively expressed in almost all human tissues and organs. In blood and immune cells, MKP‐1 is a central factor in innate and adaptive immunity^18^ and participates in monocyte adhesion and migration, macrophage proliferation/activation, and inflammatory responses.^19^, ^20^ In the nervous system, MKP‐1 is associated with the survival and death of neurons, glial cell function, learning, and memory.^21^ It also plays a neurological protective role in degenerative diseases, including AD^22^ and Huntington's disease. Within cells, MKP‐1 functions as a dephosphorylase of pTyr and pSer residues within several members of the MAPK superfamily of proteins^23^ also comprised of extracellular signal‐regulated kinase 1/2 (ERK), c‐Jun N‐terminal kinases (JNK), and p38 kinases (p38).^24^ Interestingly, the well‐documented activation of certain MAPKs seemingly correlates with some aspects of AD pathogenesis^25^ with MKP‐1 being implicated as a negative regulator.^23^ In a previous study, we demonstrated that MKP‐1 expression is downregulated in AD brains in a time‐dependent manner: the reduction of MKP‐1 expression affects Aβ generation in neurons.^22^ In addition, we found that MKP‐1 is also expressed in microglia, and we hypothesized that microglia‐sourced MKP‐1 participated in the pathogenesis of AD.

The specific aim of the present study was to explore MKP‐1 expression within microglia of AD brains and determine how this might alleviate or impede the pathological process of AD by manipulation of the M1 to M2 microglia phenotypic flux that favors the M2‐state to thereby re‐modulate neuroinflammation. 12‐month‐old mice and BV2 microglia were selected for immunofluorescence observations, immunoblotting, real‐time quantitative polymerase chain reaction (RT‐qPCR), and enzyme‐linked immunosorbent assay (ELISA) to investigate the relationship between MKP‐1, microglia, and neuroinflammation. The outcomes of this study provide novel insights and prospects for the clinical treatment of AD.

## 2 MATERIALS AND METHODS

### 2.1 Animals

APP/PS1 double transgenic (AD model) mice (APPswe, PSEN1de9) were purchased from Nanjing University‐Nanjing Institute of Biomedical Research (Certificate of Conformity No. 201803104; 201806178), bred, and genetically identified by PCR using mouse tail DNA and determined to be eligible for use. Male AD model mice and C57BL/6J mice wild‐type (WT) were selected for this study. The mice were bred and maintained at the Animal Experimentation Centre of Chongqing Medical University (12 h light/dark cycle) with free access to fodder and water. All animal experimental procedures were conducted with the approval of the Animal Ethics Committee of Chongqing Medical University and in accordance with the related regulations on animal testing and research ethics. Animal experiments were performed in a blinded manner, and the experimenter who performed the data analysis was blinded to the groups of mice at all times.

### 2.2 Immunofluorescence staining

The cryosections were rewarmed at 37°C for 1 h, and washed for 15 min to remove impurities with 1× phosphate buffered saline. The brain sections were incubated with blocking reagents (Beyotime Biotechnology) for 1 h, followed by overnight incubation with primary antibodies at 4°C. The next day the corresponding secondary antibodies (1:300, Proteintech) were incubated at 37°C for 1 h in the dark. Sections were stained with 4′,6‐diamidino‐2‐phenylindole (DAPI) and mounted with anti‐fluorescence quenching sealer after a thorough wash. The samples were visualized using a fluorescence microscope (Leica Instruments). The following antibodies of immunofluorescence detection were used in this study: anti‐Iba1 (1:100, GT10312, Genetex), anti‐Iba1 (1:100, DF6442, Affinity Biosciences), anti‐MKP‐1 (1:100, AF5286, Affinity Biosciences), anti‐amyloid β‐protein (1:100, NBP2‐13075, NOVUS), anti‐Arg1 (1:100, 16,001‐1‐AP, Proteintech), and anti‐iNOS (1:100, AF0199, Affinity Biosciences).

**FIGURE 1:**
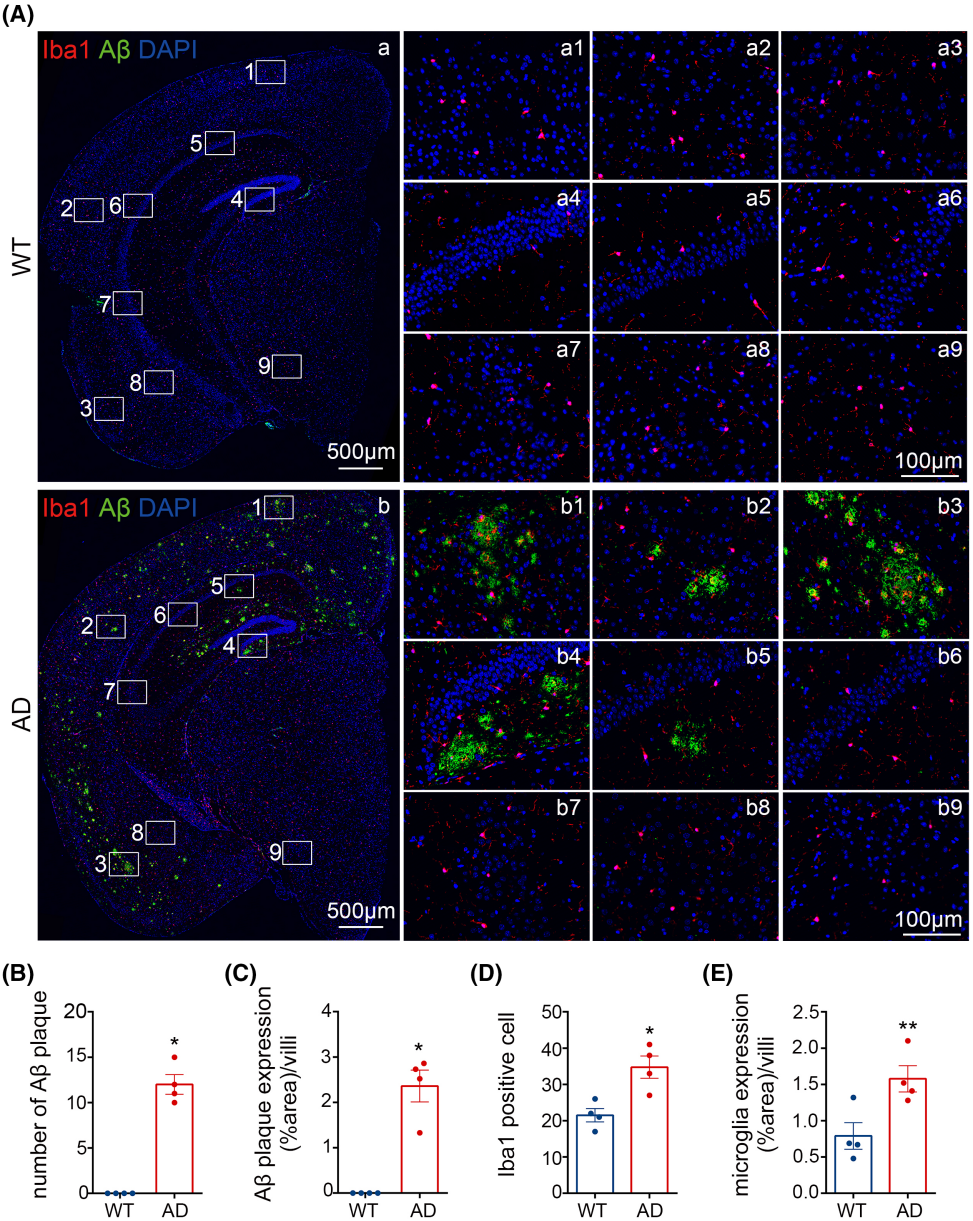
The expression of microglia and amyloid‐β (Aβ) plaques in wild‐type (WT) and Alzheimer's disease (AD) mice brains. (A) The distribution of microglia and Aβ plaques in mice brains. (a1, b1) Visual cortex, (a2, b2) auditory cortex, (a3, b3) pear‐shaped cortex, (a4, b4) DG, (a5, b5) CA1, (a6, b6) CA2, (a7, b7) CA3, (a8, b8) amygdala, and (a9, b9) hypothalamus. Iba1 (red) for microglia, 4G8 (green) for Aβ, DAPI (blue) for nuclei. Scale bar: a–b, 500 μm; a1–a9, 100 μm; b1–b9, 100 μm. (B–E) The number and area ratio of microglia and Aβ plaques were detected. The results are presented as the mean values ± SEM, *n* = 4 per group. *p < 0.05, **p < 0.01.

**FIGURE 2:**
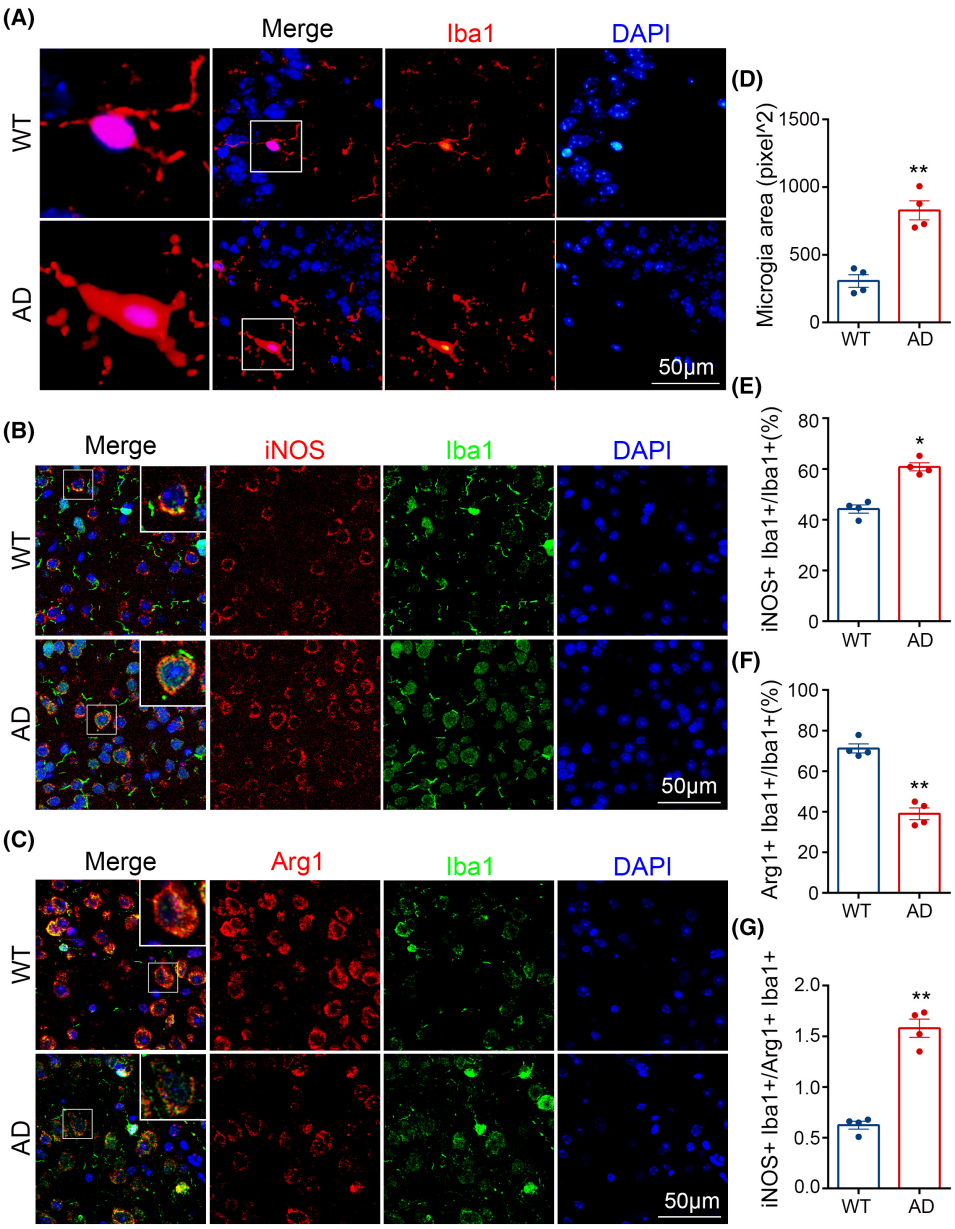
Morphological and phenotypic changes of microglia in the brain of Alzheimer's disease (AD) mice. (A) Immunofluorescent labels were performed in mice brains. Iba1 (red) for microglia, 4′,6‐diamidino‐2‐phenylindole (DAPI) (blue) for nuclei. Scale bar: 50 μm. (B, C) Immunofluorescence double‐labeling was conducted in the brain of wild‐type (WT) and AD mice. Arg1 (red) was used for M2 microglia, inducible nitric oxide synthase (iNOS) (red) for M1 microglia, Iba1 (green) for total microglia, and DAPI (blue) for nuclei. Scale bar: 50 μm. (D) Microglia cytosolic area was measured using image j. (E–G) Detecting the ratio of iNOS+ Iba1+/Iba1+, Arg1+ Iba1+/Iba1+, and iNOS+ Iba1+/Arg1+ Iba1+ with image j. The results are exhibited as the mean values ± SEM, *n* = 4 per group. *p < 0.05, **p < 0.01.

**FIGURE 3:**
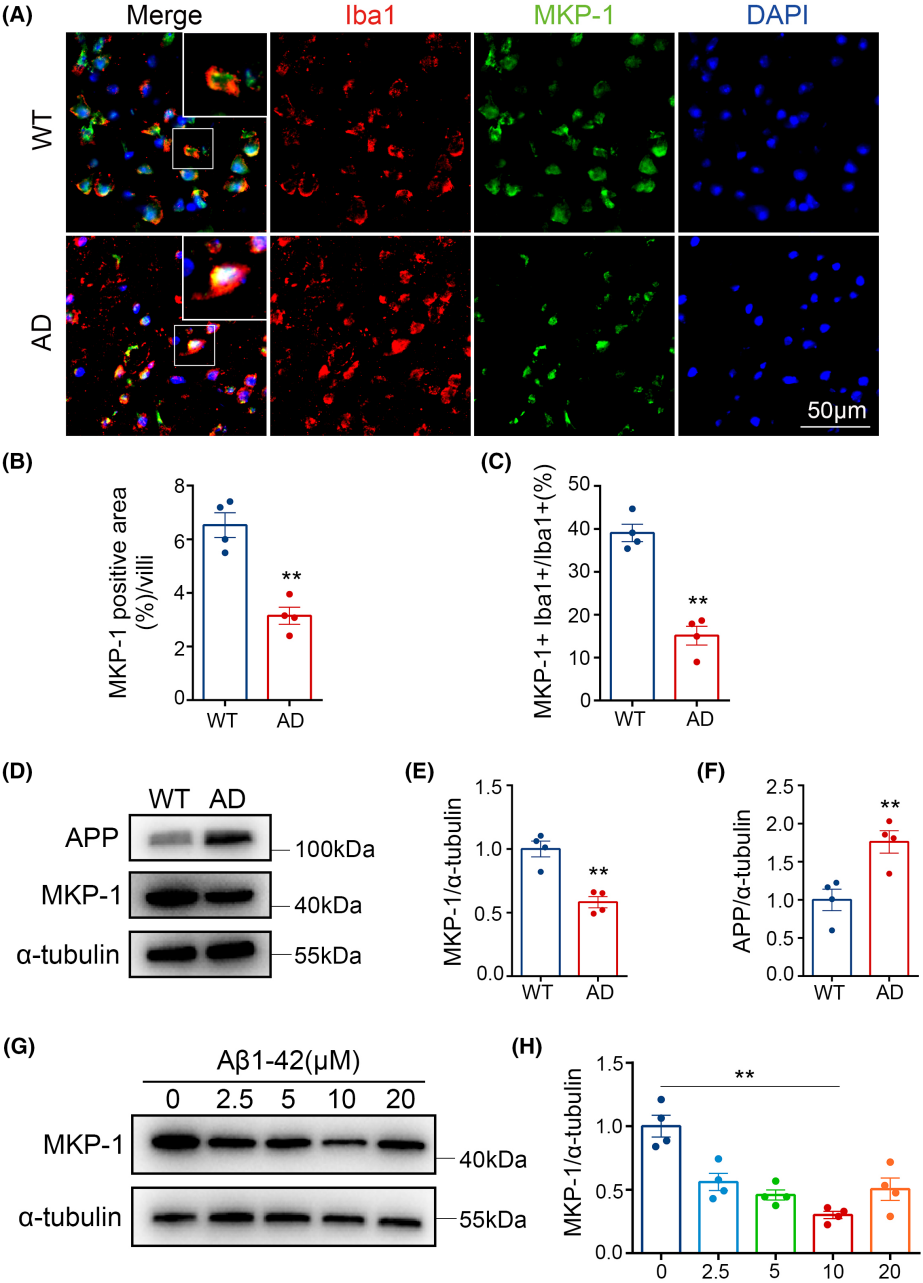
MKP‐1 expression lowered in Alzheimer's disease (AD) model mice and AD model cells. (A) Immunofluorescence staining was performed in the brain of wild‐type (WT), AD mice. Iba1 (red) for microglia, green for MKP‐1, and 4′,6‐diamidino‐2‐phenylindole (DAPI) (blue) for nuclei. Scale bar: 50 μm. (B, C) Testing MKP‐1 positive area (%) and MKP‐1+ Iba1+/Iba1+ ratio in WT and AD mice. (D–F) Immunoblotting examined MKP‐1 and amyloid precursor protein (APP) protein levels in the brains of WT and AD mice. (G, H) Western blot analyses of BV2 microglia cell lines treated with amyloid‐β (Aβ) in a gradient level of 0, 2.5, 5, 10, and 20 μM. The results are displayed as the mean values ± SEM, *n* = 4 per group. *p < 0.05, **p < 0.01.

For each mouse, we chose three sections, each section with 3 randomly selected views, and the average of the data from the selected was the data for each mouse. When acquiring the images, we chose 10 × 40 for Figures 2 and 3 to analyze but for clearer display we intercepted the magnified part to present in the text. For Figure 1, we scanned the stained sections, randomly captured 10 × 10 images for analysis of Aβ and microglia, and truncated enlarged fluorescence images of the corresponding regions for demonstration.

The percentages of positive area of Aβ plaques, IBA1 and MKP‐1; the number of positive Aβ plaques and microglia; the cytosolic area of microglia; the separate proportion of M1, M2 microglia and MKP‐1 to total microglia and the ratio of M1/M2 microglia were analyzed by image j, respectively.

### 2.3 Plasmid construction

The gateway method was used to construct the MKP‐1 overexpression plasmid. The consensus coding sequence region of mouse MKP‐1 was amplified by PCR. The PCR products were gel purified and inserted into the gateway PDNOR221 vector plasmid and then cloned into the pcDNA3.1 vector (pcDNA3.1‐CMV‐MCS‐3flag‐zsGreen) using the BP and LR reactions. The MKP‐1 gene on the vector was sequenced and confirmed.

### 2.4 Aβ oligomer preparation

The Aβ_1‐42_ peptide was purchased from Nanjing TGPeptide Biotechnology, 1 mg dissolved in 220 μL of 5% ammonia and configured to a concentration of 1 mM for subsequent experimentation.

### 2.5 Cell transfection and treatment

BV2 cells were cultured in complete medium (containing 90% DMEM, 10% fetal bovine serum (FBS) supplemented with 100 U/mL penicillin and 100 μg/mL streptomycin) and passaged every 2 days. To explore the effect of Aβ on MKP‐1, we set the concentration gradients of Aβ as 0, 2.5, 5, 10, and 20 μM for 24‐h treatment,^22^ and finally selected one concentration for subsequent experiments. Then, to further examine the effect of MKP‐1 on microglia, MKP‐1 overexpression plasmids were transfected into BV2 cells. Post 6–12 h after plating, the plasmid mixture was prepared according to the lipo3000 (Invitrogen) instructions and added to the six‐well plate. Transfected cells were placed at 37°C in a 5% CO_2_ atmosphere and incubated for 6 h with medium free of FBS and antibiotics. The medium was then changed to complete medium and incubated for 24–48 h under the above conditions, with Aβ (10 μM) treatment 24 h prior to cell collection. The control group was transfected with an overexpression of negative control plasmid and 5% ammonia water was added 24 h prior to cell collection. In order to inhibit MKP‐1 expression, we chose a 1 μM concentration of triptolide to perform the experiment. We treated the cells with Aβ (10 μM) for 24 h, and then 1 μM triptolide was added 6 h prior to cell collection. The solvent control group was added with dimethyl sulfoxide and 5% ammonia. Cells were harvested to extract RNA or protein samples and subjected to PCR or WB assays for the target of interest.

### 2.6 Cell viability assay

Cell viability was tested by cell counting kit‐8 (CCK8, MCE) assay. Cells were inoculated into 96‐well plates and relevant cell treatments or transfections were performed, then 10 μL CCK8 solution was added to each well, incubated at 37°C, and the optical density value at 450 nm was measured.

### 2.7 Western blot assay

To detect the protein levels of the microglial MAPK pathway in BV2 cells after treatment, samples were homogenized, and the cell protein extract was prepared using RIPA (Beyotime Biotechnology) and PMSF (Beyotime Biotechnology) as instructed. The protein concentration was determined using a BCA protein concentration kit (Beyotime Biotechnology). All proteins were boiled with working SDS loading buffer at 95°C for 10 min. Equal amounts of proteins were separated on sodium dodecyl sulfate‐polyacrylamide gel electrophoresis and transferred onto PVDF membranes (Millipore). After blocking by using 5% nonfat milk at room temperature for 2 h, the target proteins were immunoblotted with primary antibody overnight at 4°C. After washing with TBST (TBS with 0.1% Tween‐20), the membranes were incubated for an hour at room temperature with horseradish peroxidase‐conjugated secondary antibody (1:5000, Proteintech). The bands were visualized using enhanced chemiluminescence reagent Western blotting substrate and then detected with the BioRad image system. Protein bands were also quantified with Image J software.

The following primary antibodies were used: anti‐MKP‐1 (1:1000, A5382, Bimake), anti‐JNK (1:1000, 9252S, Cell Signaling Technology (CST)), anti‐P‐JNK (1:1000, 4668S, CST), anti‐ERK (1:1000, 4695S, CST), anti‐P‐ERK (1:1000, 4370S, CST), anti‐P38 (1:1000, 8690S, CST), anti‐P‐P38 (1:1000, 4511S, CST), anti‐amyloid precursor protein (1:5000, ab32136, Abcam), and anti‐α‐tubulin (1:5000, 11,224‐1‐AP, Proteintech).

### 2.8 RNA isolation and RT‐qPCR

Total RNA was extracted from the BV2 cells using TRIzol reagent (Tiangen), and the concentration and purity were tested with a spectrophotometer Nano‐500 (All Sheng). One microgram of RNA was reverse transcribed into the first‐strand complementary DNA (cDNA) with the Prime Script RT reagent Kit with gDNA Eraser (TaKaRa). RT‐qPCR analysis of cDNA samples was performed using TB Green Premix Ex Taq II (TaKaRa). The cDNA samples were tested in triplicate with the CFX Manager software detection system (Bio‐Rad). The sequences of the primers used are detailed in Table 1.

**TABLE 1:** Primer sequences for GAPDH, MKP‐1, Arginase 1 (Arg1), triggering receptor expressed on myeloid cells 2 (Trem2), inducible nitric oxide synthase (iNOS), and tumor necrosis factor‐alpha (TNF‐α).

Gene	Forward primer	Reverse primer
MKP‐1	AACGTCTCAGCCAATTGTCCTAA	CCTGGCAATGAACAAACACTCTC
Arg1	ACATTGGCTTGCGAGACGTA	ATCACCTTGCCAATCCCCAG
Trem2	AGTCCTTGAGGGTGTCATGT	CTTCAGAGTGATGGTGACGG
iNOS	ACCATGAGGCTGAAATCCCA	TCCACAACTCGCTCCAAGAT
TNF‐α	ACGGCATGGATCTCAAAGACA	GTGAGGAGCACGTAGTCGG
GAPDH	TGTTTCCTCGTCCCGTAGA	ATCTCCACTTTGCCACTGC

### 2.9 ELISA

The supernatants of treated BV2 cells were gathered by centrifugation at 3000 rpm/min for 10 min and the levels of mouse interleukin‐4 (IL‐4), interleukin‐6 (IL‐6), interleukin‐10 (IL‐10), and interleukin‐1β (IL‐1β) were detected by ELISA kits. Samples were measured at 450 nm using an enzyme marker.

### 2.10 Statistical analysis

The data were expressed as mean ± SEM using Graph Pad Prism 8 software. The Shapiro–Wilk test was used to test the normality of the data distribution. When the data conformed to a normal distribution, the unpaired *t*‐test was used between two groups, the one‐way ANOVA followed by Tukey's multiple comparisons test was employed between multiple groups, and the Mann–Whitney test was used when the data did not comply with a normal distribution. All of the data conform to a normal distribution, except for the number of Aβ plaques and the proportion of the positive area for Aβ plaques in Figure 1. *p* < 0.05 were considered to be statistically significant. The data for all experimental analyses were duplicated at least 3 times and the results were coincident. Also, the laboratorians who conducted the statistical analyses were kept blinded to the groups of animals and cells throughout.

## 3 RESULTS

### 3.1 Differential regional distribution of microglia and Aβ

To explore the distribution of microglia and Aβ, we performed immunofluorescence labeling of Iba1 and Aβ in 12‐month WT and AD mice (Figure 1). We observed that Aβ plaques were mainly distributed in cortical and hippocampal regions in AD mice brains, with the highest number of morphologically large Aβ plaques present in both the visual, pear‐shaped cortices and hippocampal DG area (Figure 1Ab1,b3,b4). Morphologically smaller Aβ plaques were present in the auditory cortex and CA1 zone of the hippocampus (Figure 1Ab2,b5), whereas plaques were unobserved in CA2, CA3, amygdala and hypothalamus regions (Figure 1Ab6–b9). The expression pattern of microglia was similar to Aβ plaques in AD. In contrast, no Aβ plaques were observed in WT brain and microglia were distributed across several subregions, including the cortex (Figure 1Aa1–a3), hippocampus (Figure 1Aa4–a7), amygdala (Figure 1Aa8) and hypothalamus (Figure 1Aa9). These immunofluorescence staining data also indicated that the number of microglia increased (*p* < 0.05, Figure 1D; *p* < 0.01, Figure 1E) and that Aβ plaques were clearly present (p < 0.01, Figure 1B,C) in AD samples, confirming that the distribution of microglia and Aβ plaques is region‐specific and that this may relate to the zonal functioning of each entity within the respective regions of AD mice brain tissue.

### 3.2 M1‐type microglia increase in AD model mice brain

Next, we assessed any morphological and phenotypic changes that might occur within microglia during AD using immunofluorescence techniques (Figure 2). In these experiments, we observed that cytosolic regions of microglia within AD mice brains were larger in comparison to the corresponding control WT mice brain samples (Figure 2A). Indeed, the area of microglia in the WT brain averaged 300 pixel^2^ but was significantly larger in corresponding AD samples (*ca* ~800 pixel^2^; *p* < 0.01, Figure 2D). These findings suggested that microglia were activated in AD brains. Furthermore, our results showed that iNOS, a key mediator of inflammation and immune response increased (*p* < 0.05, Figure 2E), while Arg1 decreased (*p* < 0.01, Figure 2F) in AD model mice than in WT mice (Figure 2B,C), and the ratio of iNOS/Arg1 (for M1/M2) increased considerably in AD (*p* < 0.01, Figure 2G). Overall, immunofluorescence studies indicated that M1 microglia were augmented, while M2 were reduced during AD pathology under the conditions described.

### 3.3 MKP‐1 expression is downregulated in microglia of AD in vivo and in vitro

In view of the data presented above, a quest for factors required or involved in the regulation of microglia phenotype was initiated since this might provide a novel basis as a clinical therapeutic approach for the treatment of AD patients. To ascertain the relationship between the expression of MKP‐1 and the morphological phenotype and abundance of microglia, we performed immunofluorescence staining of brains from WT and AD mice in vivo, for the presence of Iba1^26^ and MKP‐1 (Figure 3). The data suggested that levels of MKP‐1 were reduced in AD mice compared with WT counterparts (*p* < 0.01, Figure 3B), specifically in microglia (*p* < 0.01, Figure 3C), and partially for Iba1 co‐expressed with MKP‐1 in both groups (Figure 3A). We also performed immunoblotting experiments to detect the protein expression in mouse brain tissue samples and observed that MKP‐1 was downregulated in AD (*p* < 0.01, Figure 3D,E). An increase in APP protein expression was employed as an assay and positive control to verify the existence of AD model mice (*p* < 0.01, Figure 3D,F).

In a separate series of control experiments in vitro, we employed the BV2 mouse microglia cell line, which was derived from c57BL/6 mice, to investigate the relationship between microglia phenotype and Aβ/MKP‐1 protein expression levels (Figure 3). To determine whether Aβ induces a change in the levels of MKP‐1, we treated BV2 cells with varying concentrations of Aβ for 24 h, then measured the cell viability and the levels of the MKP‐1 (Figure 3G). We noted that MKP‐1 expression was inversely proportional to increasing Aβ concentration, being lowest at 10 μM but was upregulated in the presence of 20 μM Aβ (*p* < 0.01, Figure 3H) and considerably lower than in the control samples. Compared with the control (0 μM), any concentration of Aβ (2.5, 5, 10, 20 μM) significantly down‐regulated MKP‐1 expression (data not shown). Also, there was no significant difference in cell viability between all the groups (Figure S1A). The benchmark for MKP‐1 expression at 10 μM Aβ was used in subsequent experimentations (see below). Overall, MKP‐1 expression in vivo and in vitro was reduced in microglia during AD, which leads to the notion of a direct link between MKP‐1 expression and microglia in AD.

### 3.4 Aβ increases M1‐type microglia and activates MAPK pathways

**FIGURE 4:**
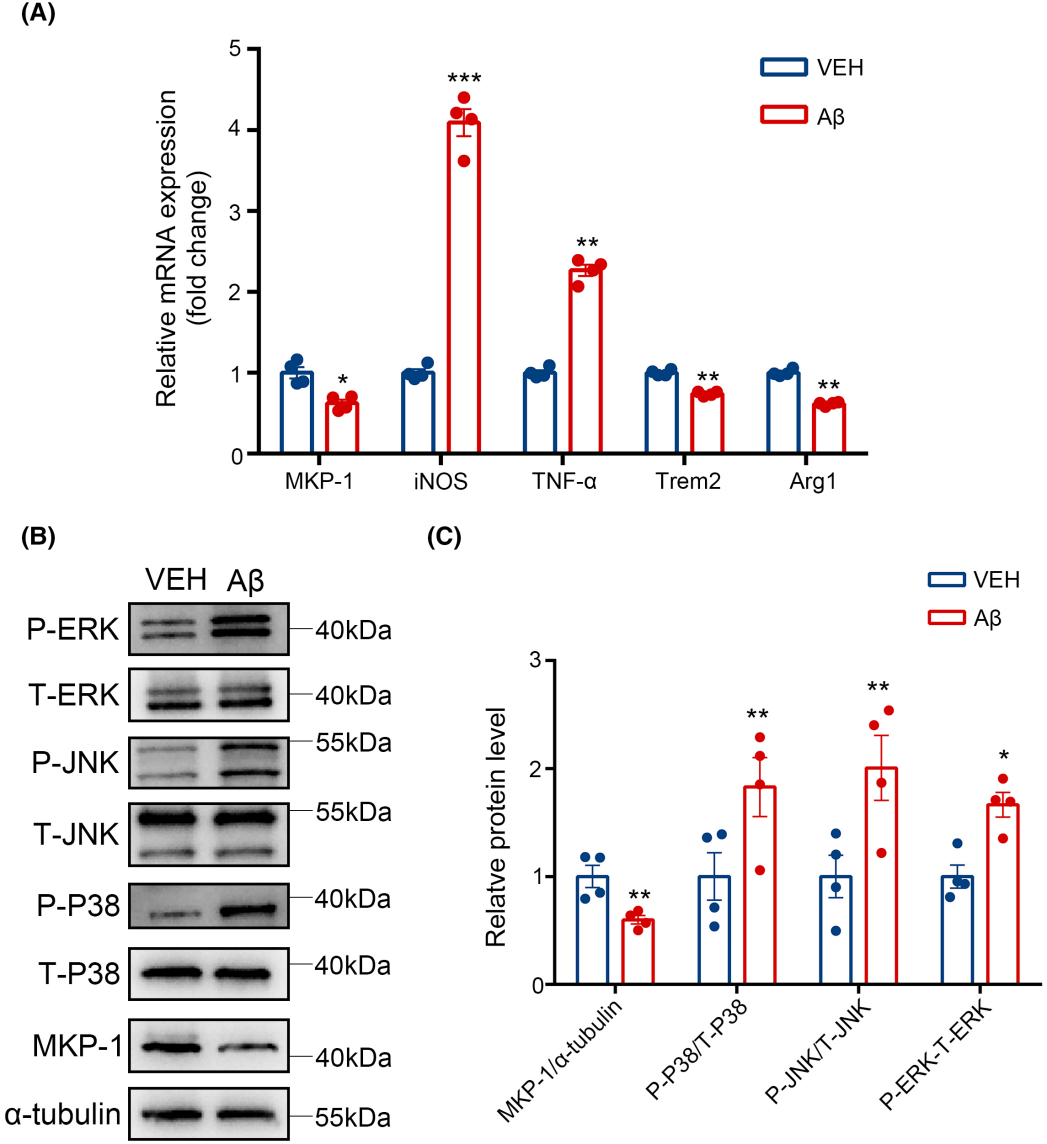
Amyloid‐β (Aβ) treatment increased M1‐type microglia and activated mitogen‐activated protein kinase pathways. (A) The mRNA levels of MKP‐1, inducible nitric oxide synthase (iNOS), tumor necrosis factor‐alpha (TNF‐α), triggering receptor expressed on myeloid cells 2 (Trem2), and Arginase 1 (Arg1) were measured after Aβ treatment using Q‐PCR. iNOS and TNF‐α were applied for M1 microglia, Trem2 and Arg1 were applied for M2 microglia. (B) Western blot to detect the protein levels of P‐ERK, T‐ERK, P‐JNK, T‐JNK, P‐P38, T‐P38, MKP‐1, and α‐tubulin after treating with Aβ. (C) The results of the western blot were quantified. The results are presented as the mean values ± SEM, *n* = 4 per group. *p < 0.05, **p < 0.01, ***p < 0.001.

In the scenario presented here, iNOS and TNF‐α were taken as representative biomarkers within M1‐state microglia, while Trem2 and Arg1 were regarded as indicative of an M2 microglial phenotype (Figure 4). We discovered that levels of iNOS (*p* < 0.001) and TNF‐α (*p* < 0.01) transcripts increased in contrast to MKP‐1 (*p* < 0.05), Trem2 (*p* < 0.01) and Arg1 (*p* < 0.01) for which levels of mRNAs were observed to decrease after treatment with Aβ (Figure 4A). We also noted that disposing of Aβ was concomitant with a decrease in MKP‐1 expression levels (*p* < 0.01), However, levels of phosphorylated (P‐) P38, P‐JNK, and P‐ERK were increased (Figure 4B,C), despite the observation that there were no detectable changes in the levels of total‐ (T‐) P38, T‐JNK, and T‐ERK. Thus, the ratio of P‐P38/T‐P38 (*p* < 0.01), P‐JNK/T‐JNK (*p* < 0.01), and P‐ERK/T‐ERK (*p* < 0.05) were evidently based on an increase in the absence or reduced levels of MKP‐1.

So Aβ aggrandized M1 microglia, lessened M2 microglia, as well as enabled ERK, JNK, and P38 pathways.

### 3.5 MKP‐1 affects microglia phenotype switching

**FIGURE 5:**
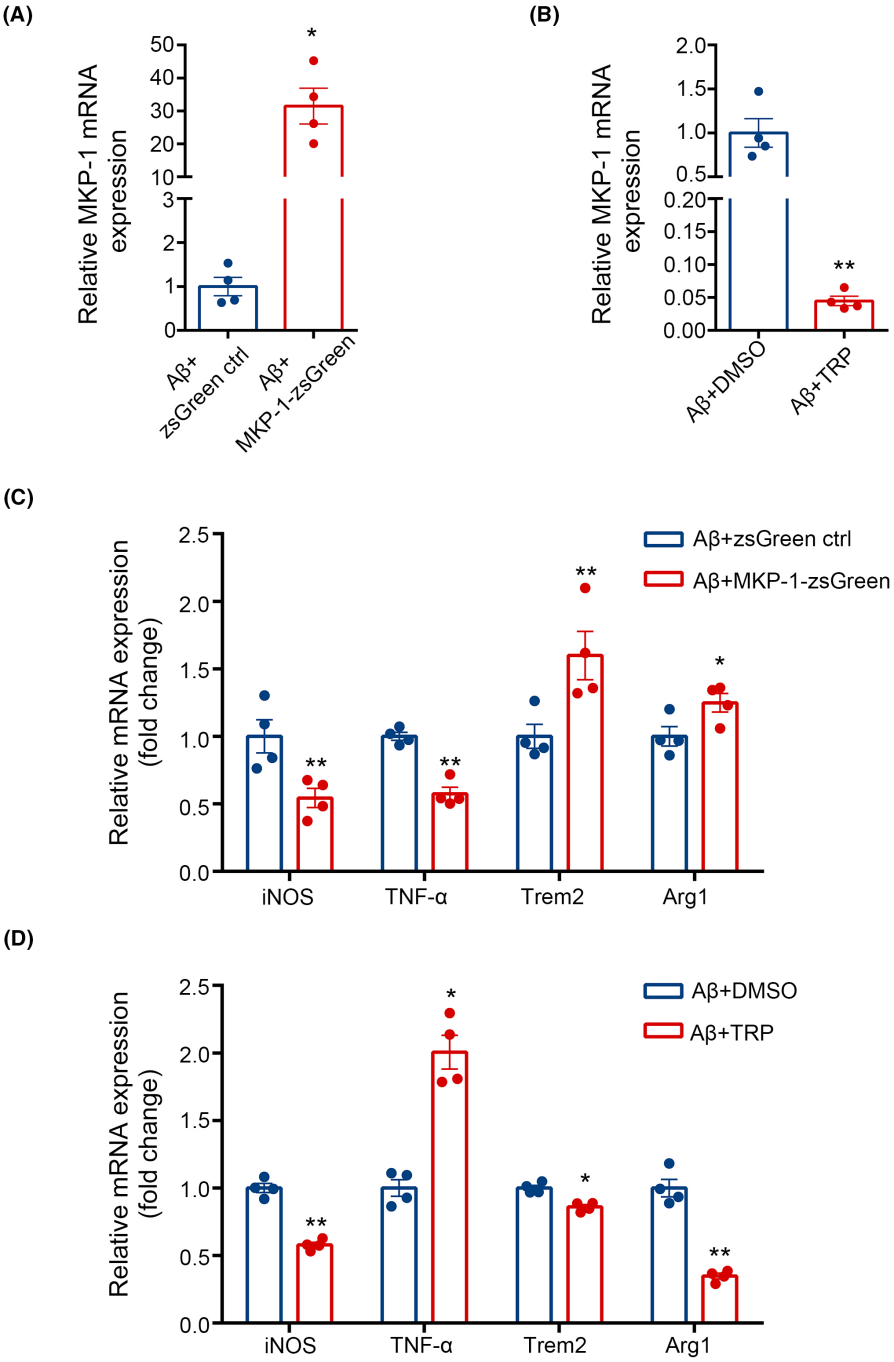
MKP‐1 regulated the M1/M2 proportion of microglia. (A, C) The detection of MKP‐1, inducible nitric oxide synthase (iNOS), tumor necrosis factor‐alpha (TNF‐α), triggering receptor expressed on myeloid cells 2 (Trem2), and Arginase 1 (Arg1) mRNA after MKP‐1 overexpression plasmid transfection. (B, D) MKP‐1, iNOS, TNF‐α, Trem2, and Arg1 mRNA level measurement after MKP‐1 inhibitor triptolide treatment. The results are shown as the mean values ± SEM, *n* = 4 for each genotype. *p < 0.05, **p < 0.01.

To further investigate whether MKP‐1 influences changes in microglia phenotype based on the Aβ treatment, we compared BV2 cells transfected with the plasmid encoding MKP‐1 or treated with the triptolide inhibitor (see materials and methods). Overexpression plasmid transfection or inhibitor treatment did not have a significant effect on cell viability (Figure S1B,C). The observations from q‐PCR assay data revealed that MKP‐1 expression was unsurprisingly upregulated during plasmid transfections and downregulated in the presence of 1 μM triptolide (*p* < 0.05, Figure 5A; *p* < 0.01, Figure 5B). However, both iNOS (*p* < 0.01) and TNF‐α (*p* < 0.01) were reduced at the level of transcription during MKP‐1 transfections, whereas mRNAs for Trem2 (*p* < 0.01) and Arg1 (*p* < 0.05) were elevated (Figure 5C). Correspondingly opposing effects were noted in the presence of triptolide, such that levels of TNF‐α (*p* < 0.05) and Trem2 (*p* < 0.05) increased but Arg1 levels were reduced (*p* < 0.01). Somewhat surprisingly iNOS (*p* < 0.01) expression was downregulated (Figure 5D). The data presented immediately above would suggest that MKP‐1 influences microglia phenotype and facilitates the transition of M1‐type microglia to M2 type. However, it remains unknown whether the changes in MKP‐1 expression contribute to additional implications.

### 3.6 MKP‐1 regulates the release of inflammatory factors from microglia

**FIGURE 6:**
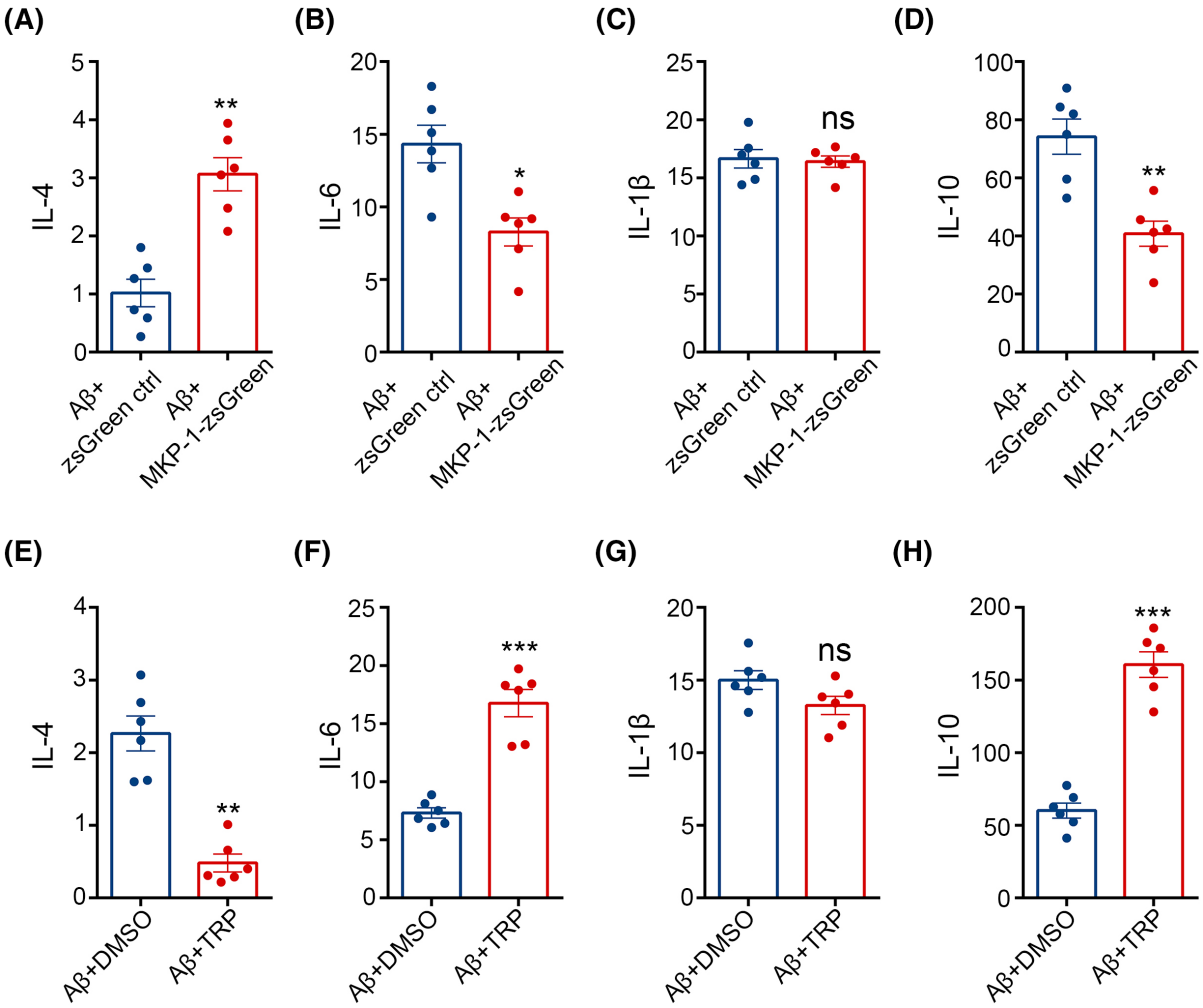
MKP‐1 adjusted the release of inflammatory factors in BV2. (A–H) The protein levels of IL‐4, IL‐6, IL‐1β, and IL‐10 were measured by ELISA after overexpressing or inhibiting MKP‐1. Cytokine levels were expressed relative to the control side. Data are expressed as mean ± SEM, *n* = 6 per group. *p < 0.05, **p < 0.01, ***p < 0.001, and ^ns^ *p* >0.05.

In view of the data presented above, it is seemingly apparent that MKP‐1 may be implicated in phenotypic conversion between M1 and M2 microglia. Thus, to further investigate the potential impact upon the secretion of inflammatory factors as a consequence of phenotypic conversion, we detected levels of inflammatory factors using ELISA (Figure 6). We found that the levels of the IL‐4 protein (*p* < 0.01) increased following transfection with the MKP‐1 expression vector, in contrast to the levels of IL‐6 (*p* < 0.05) and IL‐10 (*p* < 0.01) which were reduced (Figure 6A,B,D). We also noted that levels of IL‐4 (*p* < 0.01) were reduced after the inhibition of MKP‐1, whereas IL‐6 (*p* < 0.001) and IL‐10 (*p* < 0.001) protein expression was augmented (Figure 6E,F,H), whilst IL‐1β express remained essentially unchanged (*p* > 0.05, Figure 6C,G). These findings indicate that MKP‐1 can induce the release of IL‐4 and inhibit IL‐6 and IL‐10 secretion without impacting levels of IL‐1β expression in BV2 microglia and that MKP‐1 influences the release of both pro‐ and anti‐inflammatory factors, which contribute to the development of AD.

### 3.7 MKP‐1 regulates microglia phenotype and inflammatory factor release through the P38 pathway

**FIGURE 7:**
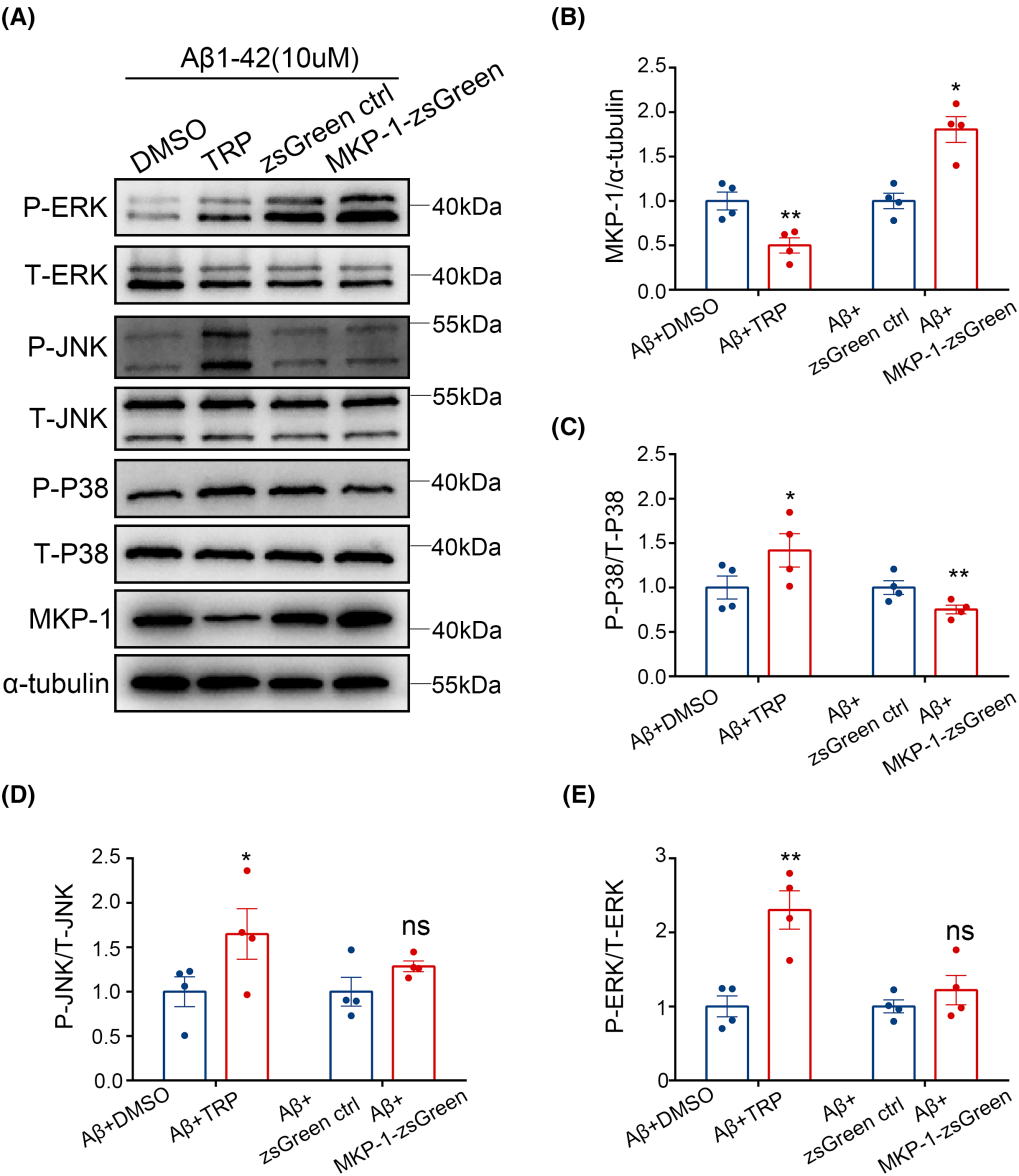
MKP‐1 worked in BV2 via the P38 pathway. (A) P‐ERK, T‐ERK, P‐JNK, T‐JNK, P‐P38, T‐P38, MKP‐1, and α‐tubulin protein levels after inhibition or overexpression of MKP‐1 using western blot. (B–E) The protein levels were quantified. Results are shown as mean ± SEM, *n* = 4 per group. *p < 0.05, **p < 0.01, and ^ns^ *p* >0.05.

To reveal the underlying mechanism(s) by which MKP‐1 regulates microglia phenotype and release of inflammatory factors, we then measured the expression levels of MKP‐1, ERK, JNK, and p38. Inhibition of MKP‐1 (*p* < 0.01, Figure 7B) resulted in a remarkable increase in P‐JNK, P‐ERK, and P‐P38, but T‐ERK, T‐JNK, and T‐P38 stabilized (Figure 7A). Therefore, P‐P38/T‐P38 (*p* < 0.05), P‐JNK/T‐JNK (*p* < 0.05), and P‐ERK/T‐ERK (p < 0.01) changed considerably after triptolide treatment (Figure 7C–E). MKP‐1 over‐expression (*p* < 0.05, Figure 7B) had a negligible impact on P‐JNK and P‐ERK, but markedly reduced the expression of P‐P38, T‐JNK, T‐ERK, and T‐P38 were essentially unchanged (Figure 7A). Hence, P‐JNK/T‐JNK and P‐ERK/T‐ERK had no significant differences, only P‐P38/T‐P38 (*p* < 0.01) substantially lowered after MKP‐1 overexpressing (Figure 7C–E). These results indicate that inhibition of MKP‐1 activates the ERK, JNK, and p38 pathway, while overexpression of MKP‐1 only inactivates p38 in BV2, so MKP‐1 may regulate microglia functions through activating or inactivating p38 pathways.

## 4 DISCUSSION

**FIGURE 8:**
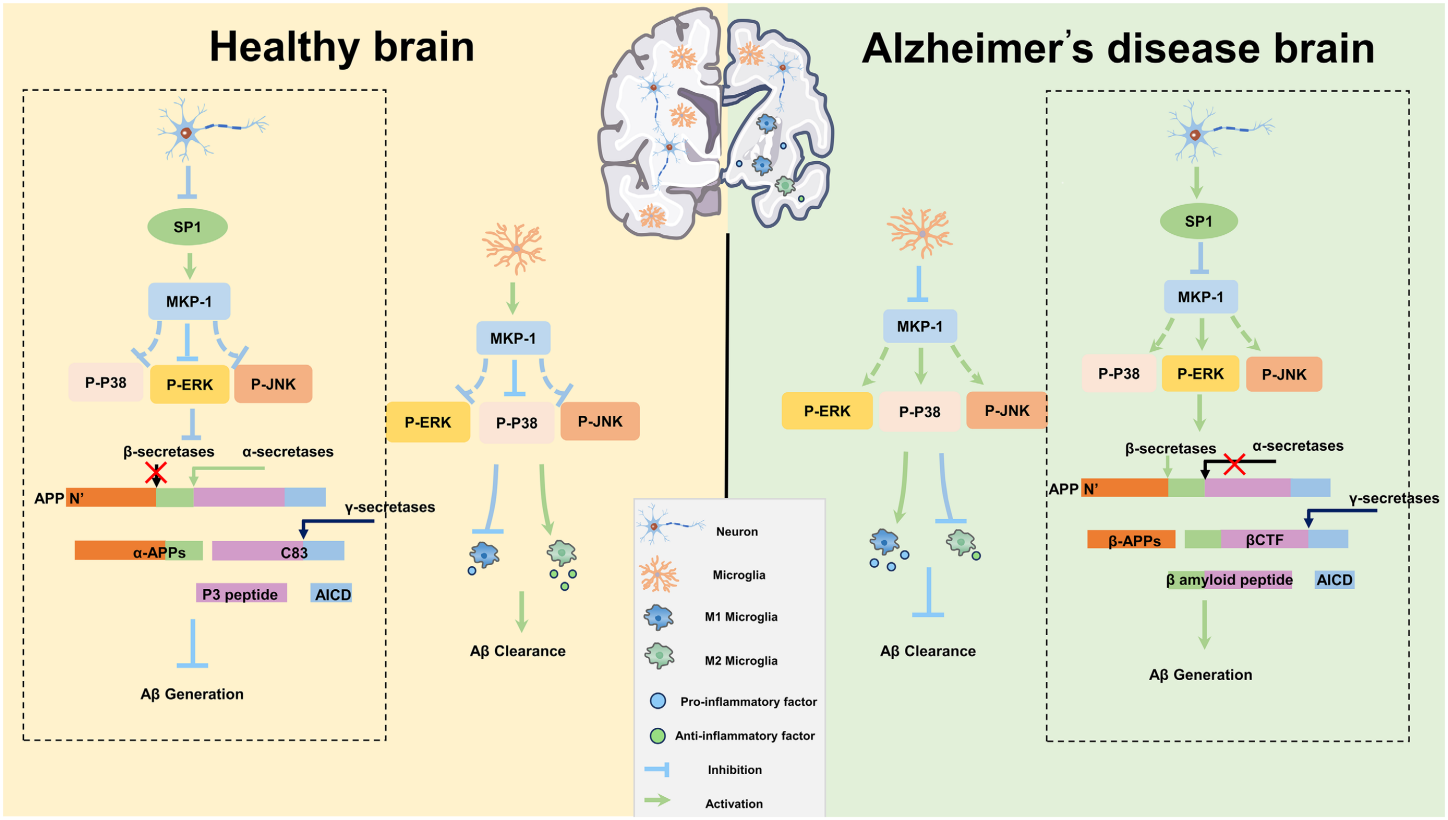
Mechanistic diagram of how MKP‐1 regulates microglia and neurons. From the perspective of microglia, downregulation of MKP‐1 activates the mitogen‐activated protein kinase pathway and increases M1 microglia and pro‐inflammatory factors; upregulation of MKP‐1 expression increases M2 microglia and anti‐inflammatory factors by inhibiting the P38 pathway. From the viewpoint of neurons, MKP‐1 inhibits the production of β‐secretases and amyloid precursor protein (APP) through the extracellular signal‐regulated kinase (ERK) pathway, further suppressing the generation of amyloid‐β (Aβ). The opposite pattern is observed in Alzheimer's disease. The parts marked with dotted lines are the content we have published.^22^

Neuroinflammation due to inflammatory activation of microglia is considered to be a potent contributor to the progression of AD. Interestingly, ever‐increasing citations suggest that secretion of pro‐inflammatory cytokines from activated microglia underlies neuronal damage,^27^ the main suspect of AD progression. Here, we have demonstrated novel and innovative insights related to microglial activation and reprogramming (Figure 8). We showed that MKP‐1 expression was downregulated in the brains of AD model mice and in AD model cells. We also found that MKP‐1 may potentially influence microglial phenotypical changes related to the release of inflammatory factors in the BV2 cell line, which may be related to the p38 pathway. In the present study, we not only observed downregulation in the expression of MKP‐1 in AD and in microglia but also in the AD model of BV2 microglia, which lead us to speculate that there may be a link between MKP‐1 and the phenotypic display of microglial cells.

Given the clinical enormity of AD development and progression, pathological changes in Aβ remain the bio hallmark of the disease,^28^, ^29^ characterized by excessive Aβ aggregation causing damage to the brain, and with the activation of microglial cells considered to be the primary route for Aβ clearance in the brain.^30^ During our study of 12‐month‐old WT and AD mice brains, we observed that Aβ was present in AD and its distribution was varied across brain regions, for example, cortical and hippocampal distributions may correlate with learning and memory dysfunction in patients with Alzheimer's. Increased numbers of activated microglia with enlarged cytosolic compartments appear to migrate proximally to Aβ plaques, which might denote clearance. Moreover, many additional studies suggest that increased numbers and size of microglia are indicative of activation.^31^ Microglia activation involves two phenotypes, each with distinct biomarker proteins. First, M1 microglia markers primarily relate to levels of CD11b, CD16, iNOS, and TNF‐α expression. Second, those within M2 microglia include Arg1, CD206, Trem2, and Ym1.^32^ In this study, TNF‐α and iNOS were selected as biomarkers within M1 microglia alongside Arg1 and Trem2 within M2 cells. Our data showed that in AD mice brains, levels of iNOS were appreciably elevated by 39% and that the iNOS/Arg1 ratio increased by at least twofold.

Moreover, levels of the amino acid homocysteine, a cellular reserve of sulfate, are increased in AD^33^ and consequently may contribute to inflammatory events.^34^ One important aspect of sulfate equilibrium relates to sulfotransferase, which is downregulated in AD,^35^, ^36^, ^37^ and thereby reduces available sulfate within the more abundant M1 microglia compared to decreased numbers of M2 microglia in which sulfate levels are deemed to be higher.^38^ In relation to neuroinflammation, it has also been recently shown homocysteine can reduce MKP‐1 levels.^39^ We also adjusted levels of MKP‐1 to monitor changes in microglia by employing MKP‐1 transfection studies to promote M1 to M2 microglial phenotype conversion. While the inhibition of MKP‐1 significantly reduced the expression of M2‐type microglia, it also influences the ratio of M1 to M2. Interestingly, inhibition of MKP‐1 also significantly influenced the expression of Arg1, Trem2, and iNOS, at the same time as increasing TNF‐α. The changes in iNOS after inhibitor treatment were not as expected, but the changes in other M1, and M2 indicators were in line with expectations. One possibility is that triptolide is not a specific inhibitor of MKP‐1, since inhibition of iNOS expression has been observed in a rat model of delayed‐onset muscle soreness.^40^ In addition, treatment with triptolide is consistent with a decrease in the expression of iNOS in lipopolysaccharide‐treated microglia.^41^, ^42^ Although confirmation of these phenomena will have to await further investigation our present study clearly demonstrated the effectiveness of MKP‐1 in the pathogenesis of AD could be harnessed as a clinical treatment strategy for AD.

M1 microglia release pro‐inflammatory factors including, IL‐1β, IL‐6, IL‐12, IL‐18, whereas M2 microglia release anti‐inflammatory factors IL‐4, IL‐10, IL‐13, IL‐33, TGF‐β.^43^ Our findings showed that not only do microglia undergo phenotypic M1/M2 conversions, but the types of inflammatory factors secreted after modulation of MKP‐1 are also affected. MKP‐1‐mediated upregulation of IL‐4, downregulation of IL‐6 and IL‐10, and inhibition of MKP‐1 represent opposing findings. Although IL‐10 is considered anti‐inflammatory, it may also be controversially pro‐inflammatory and the dual role has not been well explained.^44^ We had initially anticipated that the upregulation of MKP‐1 would be accompanied by a downregulation of IL‐1β, however, we observed a statistically insignificant result in the levels of IL‐1β (Figure 6C,G), but the results for IL‐4, IL‐6 could prove our successful treatments. Indeed, with little notable change in the levels of IL‐1β expression within BV2 cells, the changes in levels of MKP‐1 expression appear unrelated to the NLRP3 pathway associated with IL‐1β. Thus MKP‐1 might influence other signaling pathways implicated in the regulation of microglial cell polarization. For example, in the toll‐like receptor (TLR) signaling pathway, microglial activation mediated through TLR4 expression,^45^ is coupled to binding interactions with LPS to facilitate M1 microglia polarization.^46^ Additionally, inhibition of the nuclear factor κ‐B pathway may influence conversion to the M2 microglial type.^47^

And MKP‐1 is a negative regulator of the MAPK pathways, a superfamily of serine–threonine protein kinases that are activated by different extracellular stimuli,^48^ presumably MKP‐1 may also regulate the phenotype and function of microglia through the same. Of these, activation of JNK plays an essential role in mouse development by moderating cell survival, apoptosis, and proliferation.^49^ In AD, p‐JNK directly mediates the formulation of NFTs and promotes the process of tangle maturation through direct phosphorylation of Tau proteins.^50^ Furthermore, ERK, also known as p42/p44 MAPK, that participates in the six essential cellular life activities, has been linked to learning memory in AD,^51^, ^52^ may promote the death of neuronal cells, and be of influence in regulating synaptic plasticity.^53^ Moreover, p38 has been associated with tau protein phosphorylation, excitotoxicity, synaptic dysfunction, and especially neuroinflammation during AD.^54^, ^55^ An increasing body of evidence suggests that the p38 signaling pathway is central to normal immune and inflammation responses.^25^, ^56^ The activation of p38 activation in the presence of Aβ also contributes to the phosphorylation of tau^57^ and facilitates the amyloidogenic processing of APP.^58^ In addition, a novel compound, VB‐037, can ameliorate neuronal damage and neuroinflammation by inhibiting the p38 signaling pathway.^59^ In conclusion, p38 participates in several aspects of AD and plays an influential role, particularly during inflammation. For instance, our results suggested that up‐ or downregulation of MKP‐1 levels in BV2 resulted in changes to the p38 pathway with a minor impact upon the corresponding JNK and ERK pathways (Figure 8), thus MKP‐1 is p38‐selective in microglia. Other aspects for consideration include siRNA transfections in which MKP‐1 was downregulated by approximately 30% and no MKP‐1‐specific inhibitor was available. Instead, a broad‐range inhibitor, triptolide was applied with a knockdown efficiency of greater than 90% in MKP‐1 levels. The difference between siRNA and inhibitors is that siRNA targets mRNA prior to protein expression, acting on upstream nucleic acids, whereas inhibitors target downstream proteins.

In summation, the results of this study explored the mechanisms by which MKP‐1 regulates AD‐related pathology and microglial cell genetic phenotype and biochemistry, thereby bridging gaps with our previous research reports concerning neurons. We have drawn a mechanistic map of the mechanisms by which MKP‐1 regulates AD‐associated pathological processes based on the results of this study and our previous work (Figure 8). Together, these provide new insights into the role of MKP‐1 in the development of AD and its possibility as a new AD therapeutic target.

## AUTHOR CONTRIBUTIONS

Guiqiong He and Kejian Wang conceived, designed, and supervised the entire study. Junhua Li and Lin Wang performed the experiments, acquired data, analyzed data, and drafted the manuscript. Qinhua Zeng participated in part of the experiments, acquired data, and analyzed data. Guiqiong He, Kejian Wang, and Qing Tang reviewed and edited the manuscript. All authors have read and approved the final manuscript.

## FUNDING INFORMATION

This study was supported by the Scientific and Technological Research Program of the Chongqing Municipal Education Commission (NO. KJCXZD2020021; KJZD‐K201900403), the CQMU Program for Youth Innovation in Future Medicine (W0044), and the General project of the Chongqing Natural Science Foundation (No. cstc2021jcyj‐msxmX0442).

## CONFLICT OF INTEREST STATEMENT

The authors declare that they have no conflict of interest.

## Supporting information


Figure S1.

## Data Availability

The data that support the findings of this study are available from the corresponding author upon reasonable request.
