# Supplementary material for: MKP‐1 regulates the inflammatory activation of microglia against Alzheimer's disease
Source: CNS Neurosci Ther. 2023 Aug 21;30(2):e14409. doi: 10.1111/cns.14409 (PMC10848084; doi:10.1111/cns.14409)
Supplement: Supplementary file 1 — Figure S1. [file CNS-30-e14409-s001.pdf]

Supplementary Materials

MKP-1 regulates inflammatory activation of microglia against Alzheimer's disease

Junhua Li[1,2#], Lin Wang[3#], Qinhua Zeng[1, 2], Jing He[1, 2], Qing Tang[1, 2], Kejian Wang[1, 2*], Guiqiong He[1, 2*]

1 Institute of Neuroscience, Basic Medical College, Chongqing Medical University, Chongqing 400016, China;

2 Department of Anatomy, Basic Medical College, Chongqing Medical University, Chongqing 400016, China;

3 Department of Basic Medicine, Chongqing College of Traditional Chinese Medicine, Chongqing, 402760, China.

[#] These authors contributed equally to this paper.

***Correspondence:**

Kejian Wang: kejianwang@cqmu.edu.cn

Guiqiong He: guiqionghe@cqmu.edu.cn; Tel: +86-023-68485763

Figure S1

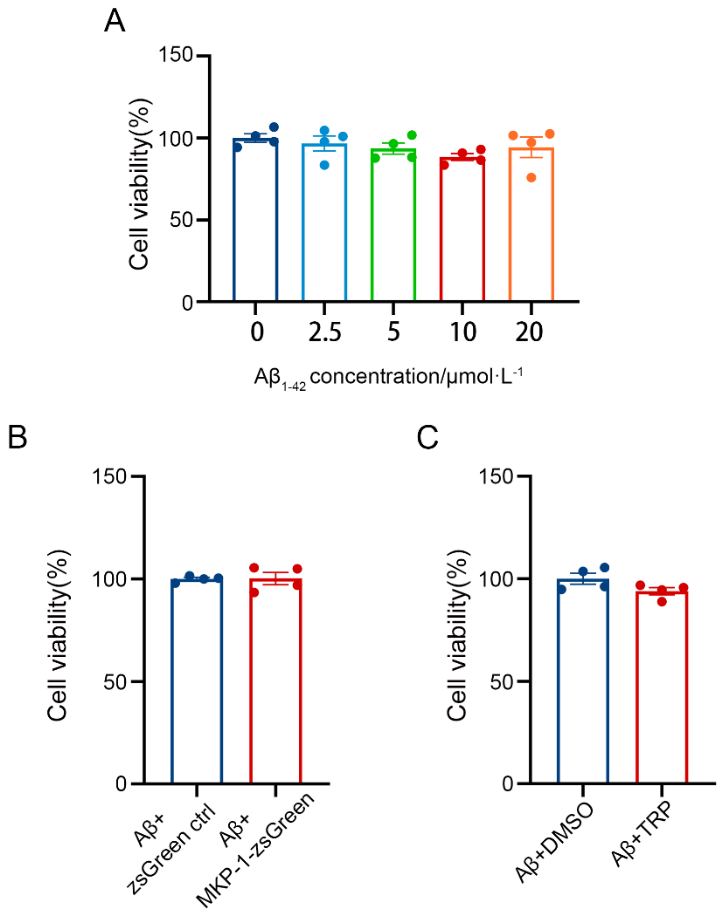

FigS1. Cell viability of all groups after treatment. **(A)** Cell viability assay of BV2 after Aβ gradient treatment. **(B)** Cell viability assay of BV2 after Aβ treatment and plasmid transfection. **(C)** Viability assay of BV2 after Aβ and triptolide treatment. The results are presented as the mean values ± SEM, n=4 per group.
